# Changes in tongue pressure and dysphagia at oral cancer patients by palatal augmentation prosthesis

**DOI:** 10.1002/cnr2.1516

**Published:** 2021-09-02

**Authors:** Izumita Kuniyuki, Takuma Hisaoka, Ryoukichi Ikeda, Jun Suzuki, Naoko Sato, Ryo Tagaino, Tomonori Kambayashi, Ai Hirano‐Kawamoto, Jun Ohta, Akira Ohkoshi, Ryo Ishii, Naru Shitraishi, Kengo Kato, Shigeto Koyama, Keiichi Sasaki, Yukio Katori

**Affiliations:** ^1^ Maxillofacial Prosthetics Clinic Tohoku University Hospital Sendai Japan; ^2^ Department of Otolaryngology and Head and Neck Surgery Tohoku University Graduate School of Medicine Sendai Japan; ^3^ Division of Advanced Prosthetic Dentistry Tohoku University Graduate School of Dentistry, Tohoku University Sendai Japan

**Keywords:** dysphagia, oral cancer, palatal augmentation prosthesis, swallowing, videofluoroscopic dysphagia scale

## Abstract

**Background:**

The palatal augmentation prosthesis (PAP) is an intraoral prosthesis used in the treatment of dysphagia.

**Aim:**

The objective of the study is to examine the effect of PAP using tongue pressure and the Videofluoroscopic Dysphagia Scale (VDS) to understand the precise mechanism for improvement in swallowing function with PAP for oral cancer at retrospective survey.

**Methods and results:**

Fifteen patients were provided PAPs. Tongue pressure and VDS were evaluated with and without PAP. After intervention with PAP, tongue pressure significantly increased as compared to when without PAP (*p* < .05). The total mean VDS score with PAP was found to have significantly improved (*p* < .05). The mean VDS score of the oral phase also significantly improved with the PAP compared to without the PAP group (*p* < .05). Significant differences (*p* < .01) were found in each category, such as tongue to palate contact and pyriform sinus residue.

**Conclusion:**

PAP can improve tongue pressure, tongue to palate contact, and pyriform sinus residue.

## 1 INTRODUCTION

**FIGURE 1:**
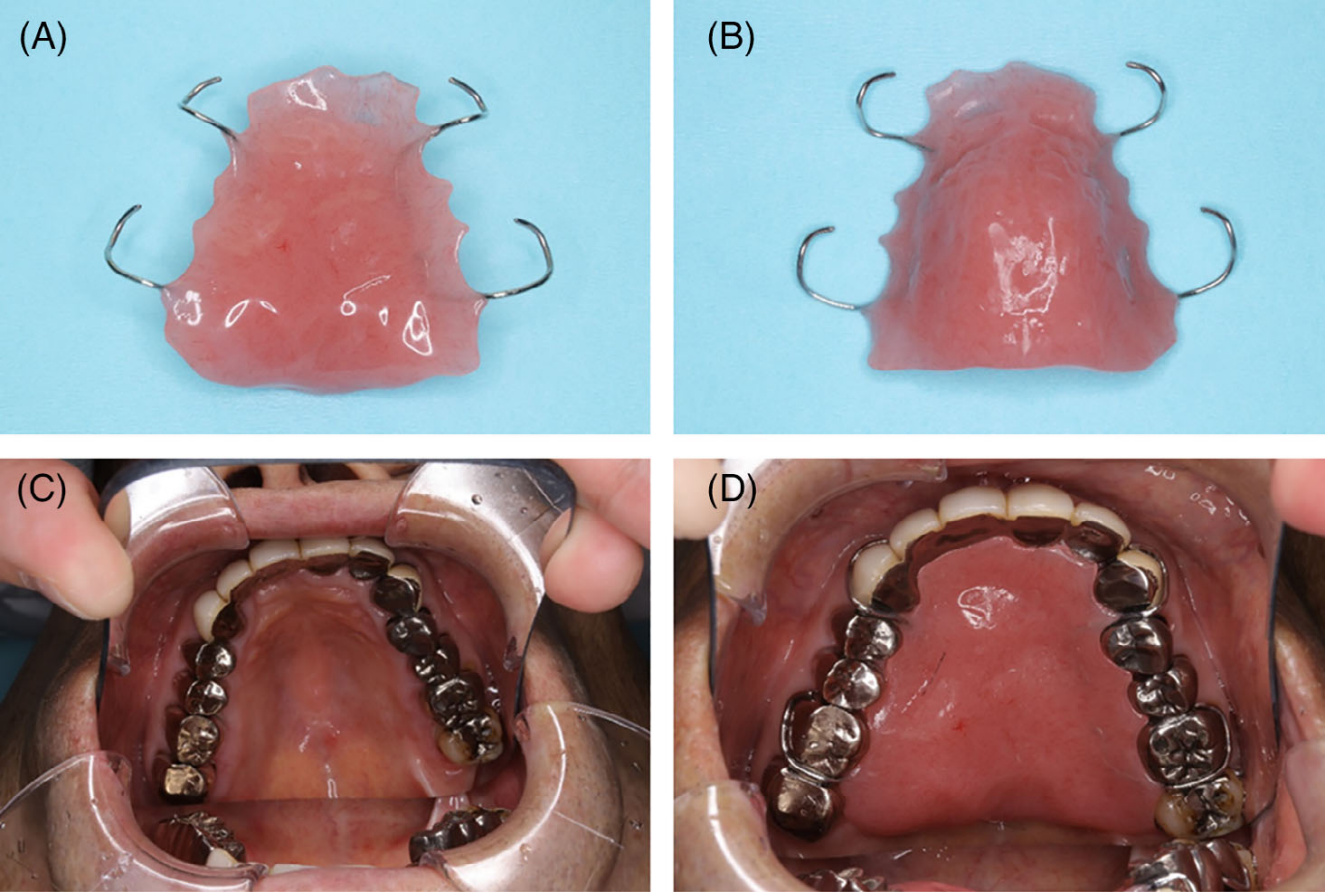
Palatal augmentation prosthesis (PAP). (A) PAP (tongue side), (B) PAP (hard plate side), (C) Without PAP, (D) With PAP

Tongue movement plays a pertinent role in swallowing. It is crucial in both maintaining the bolus as a cohesive unit through its manipulation during mastication, and propelling the bolus out of the oral cavity and through the pharynx. Tongue pressure against the hard palate is the most significant oral pressure in the propulsion of the bolus from the oral cavity and into the pharynx.^1^, ^2^ Tongue contact with the alveolar ridge and central groove exhibited centripetal and subsequent centrifugal motion that created an oropharyngeal propulsive chamber in conjunction with the pharyngeal walls.^3^ Higher tongue pressure decreases oral residue. Tongue pressure contributed to the propulsion of the food bolus from the oral cavity into the pharynx in the elderly.^4^ Tongue pressure is an indicator of other swallowing‐related muscles. Tongue‐pressure resistance training improves tongue pressure and PAS score.^5^ The older adult had lower tongue strength generally took longer to eat a meal and ate less than those with higher tongue strength.^6^ Tongue pressure has been reported to be a good predictor of the presence of dysphagia, is associated with aspiration.^7^ The palatal augmentation prosthesis (PAP) is an intraoral prosthesis used in the treatment of dysphagia (Figure 1). It allows reshaping of the hard palate to improve contact between tongue and palate during swallowing because of impaired tongue mobility. Several studies have been reported regarding PAP insertion for oral cancer patients who have undergone glossectomy.^8^ However, the detailed mechanism with which PAP improves bolus propulsion and pharyngeal function in oral cancer patients remain unknown. The Videofluoroscopic Dysphagia Scale (VDS) can predict the long‐term prognosis of dysphagia patients.^9^ It consists of 14 items, with a total of 100 points, representing oral and pharyngeal functions that can be observed by videofluoroscopic (VF) evaluation. This study aims to examine the effect of PAP on oral cancer, using tongue pressure and VDS to understand the components of swallow efficiency that PAP impacts.

## 2 MATERIALS AND METHODS

### 2.1 Patient selection

A retrospective case series was conducted in accordance with the Helsinki Declaration and was approved by the Tohoku University Hospital Institutional Review Board (IRB) (Reference number: 2014‐1‐274). All patients have received treatment, including surgery, radiation therapy, or chemotherapy, for advanced head and neck cancer (HNC).

A multidisplinary team is in charge of dysphagia cases in our hospital. This team is part of our hospital's Swallowing Centre, that was established through the collaboration between our medical and dental departments. From our database, we extracted information of the patients who had previously received HNC surgery as well as prosthetic treatment between March 2016 and March 2020. Among them, 15 patients (10 males and 5 female subjects aged 29–82 years; median 65.5 ± 12.1 years) were provided PAPs by prosthodontists.

### 2.2 Assessment of tongue pressure

Tongue pressure was measured as the maximum voluntary tongue pressure against the palate using a commercial device (JMS tongue pressure measuring instrument, JMS, Hiroshima, Japan)^10^ with or without PAP on the same day as the VDS. The instrument was calibrated to 0.0 kPa after applying pressure (19.6 ± 1.0 kPa) to balloon outside the oral cavity. Patients were placed in a sitting position, then asked to place the balloon in their mouth and hold the plastic pipe at the midpoint of their central incisors with their lips closed. The participants were instructed to compress a small balloon, attached to the probe's tip, between the tongue and the hard palate's anterior part for 7 s with maximum voluntary effort. The pressures were measured three times, and the average value was recorded.

### 2.3 Assessment of VDS

The patients were directed to swallow 3 and 5 ml of diluted barium. Subsequently, identical tests were repeated using foods such as yogurt, puddings, rice porridge, and rice with standardized viscosity and quality. The reference diet was pudding. All study procedures were recorded on AVI files (30 frames/s). After all patients finished the VFSS study, the video recordings were collected, and each file was given a random number. These files were then copied to 10 DVDs, with each DVD containing all video recordings in a different randomized order. These DVDs were sent to an interpreter for analysis. Two physiatrists analyzed the AVI files. Conclusions were drawn by consensus.

VDS consists of the oral phase and the pharyngeal phase (Table 1) based on VF results. The VF data was randomized, and blinded evaluation of data was conducted by three experienced otolaryngologists.

**TABLE 1:** The items of the videofluoroscopic dysphagia scale (VDS)

Parameter	Coded value		Score	Parameter	Coded value		Score
Lip closure	Intact	0	4	Triggering of pharyngeal swallowing	Normal	0	4.5
	Inadequate	2			Delayed	4.5	
	None	4		Vallecular residue	None	0	6
Bolus formation	Intact	0	6		<10%	2	
	Inadequate	3			10%–50%	4	
	None	6			>50%	6	
Mastication	Intact	0	8	Laryngeal elevation	Normal	0	9
	Inadequate	4			Impaired	9	
	None	8		Pyriform sinus residue	None	0	13.5
Apraxia	None	0	4.5		<10%	4.5	
	Mild	1.5			10%–50%	9	
	Moderate	3			>50%	13.5	
	Severe	4.5		Coating on the pharyngeal wall	No	0	9
Tongue to palate contact	Intact	0	10		Yes	9	
	Inadequate	5		Pharyngeal transit time	≤1.0 s	0	6
	None	10			>1.0 s	6	
Premature bolus loss	None	0	4.5	Aspiration	None	0	12
	<10%	1.5			Supraglottic penetration	6	
	10%–50%	3			Subglottic aspiration	12	
	>50%	4.5		Pharyngeal phase			60
Oral transit time	≤1.5 s	0	3				
	>1.5 s	3					
Oral phase			40	Total			100

## 3 STATISTICAL ANALYSIS

Wilcoxon signed‐rank test was performed using the statistical software SPSS version 27 (IBM, Chicago, IL). Differences with a corrected *p*‐value of less than .05 were considered significant.

## 4 RESULT

### 4.1 Patients' characteristics

A total of 15 patients (10 men and 5 women) were included in this study, with an age range of 29–82 (median 65.4 ± 11.7) years (Table 2). Their baseline characteristics are shown in Table 1. All patients had oral cancer, in which tongue cancer was the most common with 12 incident patients. The seventh edition of UICC (Union for International Cancer Control) TNM classification was applied. Since 13 of 15 were cases with advanced cancer, most had received surgery with free flap reconstruction, except for two cases with marginal mandibulectomy and with partial glossectomy. Eight cases had undergone total or subtotal glossectomy. Resection of the mandible affected mastication, dribbling of food from the oral cavity, and delayed oral transit.^11^ So we used PAP at segmental mandibulectomy cases. Fourteen patients received neck dissection (11 bilateral and 3 unilateral). No patients had a radiation history before the surgical treatment, while five patients received adjuvant (chemo)radiation therapy postoperatively. Only one patient, who had received surgery with free flap reconstruction for tongue cancer, received chemoradiotherapy for hypopharyngeal cancer after 3 years. No other patients had another duplicated cancer in the head and neck region. The period between the surgical treatment and the prosthetic treatment was varied across patients (from 6 months to 11 years). Since the VF evaluation is performed after the PAP adjustment is completed, the period before and after wearing varies depending on the case (from 0 months to 11 months).

**TABLE 2:** The patients' characteristics (First VF means the period from primary treatment to swallowing center consultation. Span means the period from primary assessment to palatal augmentation prosthesis assessment)

Sex	Age	Primary	TMN	Stage	Surgical field	Neck dissection	Free flap reconstruction	Chemoradiation therapy	First VF	Span
M	29	Tongue	T3N1M0	III	Subtotal glossectomy	Bilateral	Y	N	3 m	0.5
M	35	Tongue	T3N0M0	III	Subtotal glossectomy	Bilateral	Y	N	1 m	5
F	56	Lower gingiva	T4aN0M0	IVa	Segmental mandibulectomy	Bilateral	Y	N	1 m	11
F	61	Tongue	T4aN0M0	IVa	Total glossectomy	Bilateral	Y	N	4 m	8
F	62	Floor of mouth	T4aN0M0	IVa	Segmental mandibulectomy	Right	Y	N	3 m	4
F	64	Tongue	T3N0M0	III	Total glossectomy	Bilateral	Y	Y	1 m	1
F	65	Tongue	T1N0M0	I	Subtotal glossectomy	Bilateral	Y	N	4 m	8
M	66	Tongue	T3N1M0	IVa	Subtotal glossectomy	Bilateral	Y	Y	8 y 7 m	5
M	67	Tongue	T3N0M0	III	Subtotal glossectomy	Bilateral	Y	N	9 y 5 m	0
M	69	Floor of mouth	T1N2cM0	IVa	Marginal mandibulectomy	Bilateral	N	Y	1 y	3
M	70	Tongue	T4aN0M0	IVa	Subtotal glossectomy and segmental mandibulectomy	Bilateral	Y	Y	1 m	2
M	74	Tongue	T2N1M0	IVa	Hemiglossectomy	Right	Y	Y	7 m	1
M	78	Tongue	T2N0M0	II	Partial glossectomy	Bilateral	Y	Y	21y	5
M	82	Tongue	T2N1M0	III	Partial glossectomy	Left	N	N	3 m	6
M	82	Tongue	T3N0M0	III	Hemiglossectomy	Right	Y	N	23y	4

### 4.2 The tongue pressure

**FIGURE 2:**
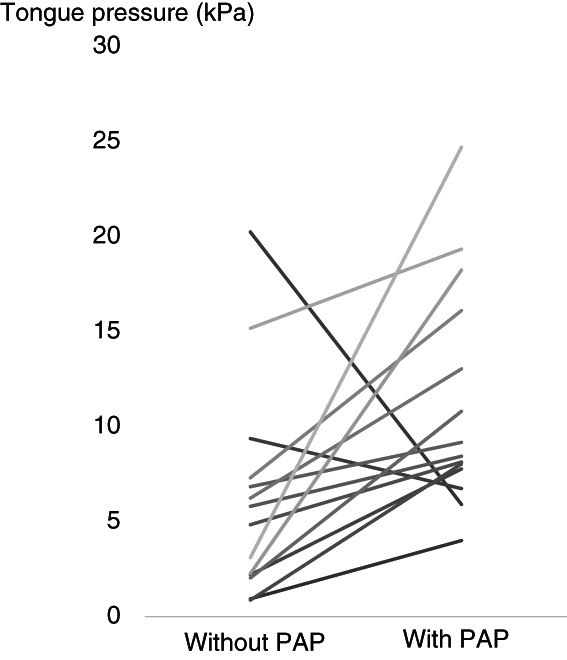
The tongue pressure without (left) and with (right) palatal augmentation prosthesis (PAP) *n* = 15

Tongue pressure was increased in 13 cases (Figure 2). Two cases decrease tongue pressure. They were cases in which outpatient adjustment was not possible and cases in which dentures were incompatible due to the appearance of new swaying teeth. After intervention with PAP, the tongue pressure was 12.3 ± 6.7 kPa, which was significantly increased compared to without PAP (6.6 ± 5.6 kPa) (*p* = .017).

### 4.3 VDS score

**FIGURE 3:**
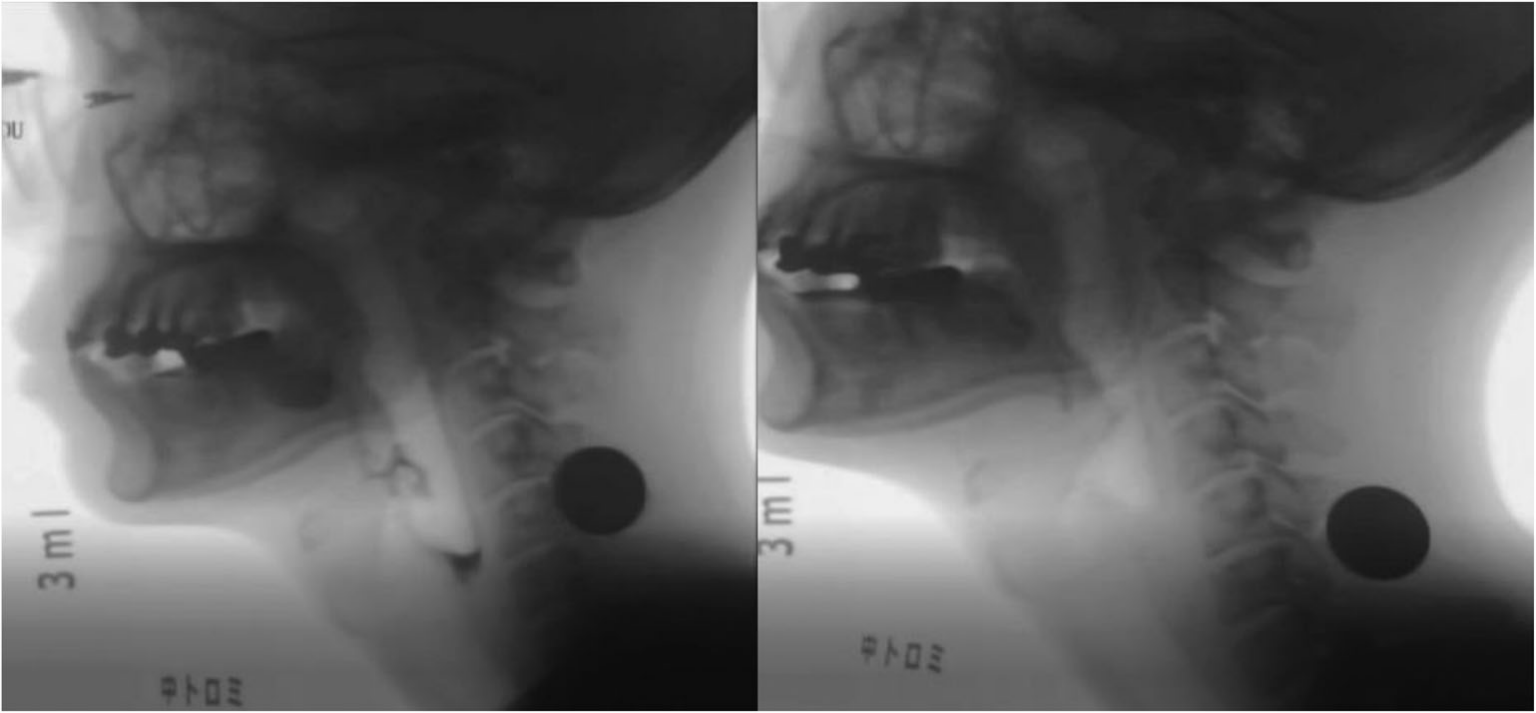
pharyngeal residue without (left) and with (right) palatal augmentation prosthesis (PAP)

The cases with improved tongue pressure (13 patients expected two patients which decrease tongue pressure) were investigated through the VDS score (Table 3). Ten patients (76.9%) had improved in their total VDS score although three patients did not improve. The total mean VDS score was 46.7 ± 19.2 and 40.1 ± 21.4 in the without and with PAP groups, respectively, which was significantly improved (*p* = .045). The mean VDS score of the oral phase also significantly improved in the PAP group (21.0 ± 6.3) as compared to the without PAP group (16.5 ± 7.5) (*p* = .015). In contrast, there was no significant difference between the two groups in the pharyngeal phase (*p* = .388). Significant differences were found in each category, such as tongue to palate contact (*p* = .010) and pyriform sinus residue (*p* = .018) (Figure 3). The CRT group had a higher VDS score without PAP. CRT does not seem to affect the improvement of VDS by PAP (Table 4). The unilateral neck dissection group tended to improve VDS by PAP (Table 5).

**TABLE 3:** The videofluoroscopic dysphagia scale (VDS) score before and after the intervention

	Before intervention	After intervention	*p*
Lip closure	0.3 ± 0.8	0.1 ± 0.2	.180
Bolus formation	3.8 ± 1.3	3.1 ± 1.5	.053
Mastication	5.2 ± 1.4	4.3 ± 2.0	.058
Apraxia	2.1 ± 0.9	1.8 ± 0.9	.605
Tongue to palate contact	5.8 ± 2.3	3.9 ± 2.6	.010*
Premature bolus loss	1.4 ± 1.4	1.4 ± 1.6	.944
Oral transit time	2.4 ± 0.8	1.9 ± 0.9	.096
Triggering of pharyngeal swallowing	2.8 ± 0.8	2.9 ± 1.4	.660
Vallecular residue	1.9 ± 1.4	2.2 ± 1.4	.131
Laryngeal elevation	6.6 ± 2.7	5.5 ± 3.3	.196
Pyriform sinus residue	4.4 ± 4.2	2.6 ± 2.9	.018*
Coating on the pharyngeal wall	4.5 ± 4.3	4.3 ± 4.1	.577
Pharyngeal transit time	2.5 ± 2.6	2.8 ± 2.3	.317
Aspiration	3.0 ± 3.9	3.6 ± 4.2	.858
Oral phase	21.0 ± 6.3	16.5 ± 7.5	.015*
Pharyngeal phase	25.6 ± 15.2	23.6 ± 17.6	.388
Total	46.7 ± 19.2	40.1 ± 21.4	.045*

*Note*: Asterisk indicated *p* < .05. *n* = 13.

**TABLE 4:** The videofluoroscopic dysphagia scale (VDS) score before and after the intervention with or without CRT

	CRT +		CRT −	
	Before intervention	After intervention	Before intervention	After intervention
Lip closure	0.1 ± 0.3	0.4 ± 1.1	0.0 ± 0.0	0.3 ± 0.7
Bolus formation	4.0 ± 1.7	4.0 ± 1.7	2.8 ± 1.3	3.6 ± 1.1
Mastication	4.9 ± 1.8	5.3 ± 1.7	4.1 ± 1.8	5.0 ± 1.1
Apraxia	1.8 ± 1.0	2.6 ± 0.7	1.9 ± 0.8	1.7 ± 0.8
Tongue to palate contact	5.0 ± 3.2	5.3 ± 3.1	3.9 ± 2.2	5.4 ± 2.3
Premature bolus loss	2.7 ± 1.5	1.9 ± 1.4	0.9 ± 1.1	1.4 ± 1.5
Oral transit time	2.2 ± 1.2	2.3 ± 0.8	1.9 ± 0.6	2.3 ± 0.9
Triggering of pharyngeal swallowing	3.8 ± 0.8	3.3 ± 1.1	2.5 ± 1.3	2.5 ± 0.6
Vallecular residue	3.1 ± 1.2	3.1 ± 1.0	1.9 ± 1.3	1.2 ± 0.9
Laryngeal elevation	8.0 ± 1.5	7.3 ± 2.0	4.3 ± 3.4	5.8 ± 2.6
Pyriform sinus residue	4.6 ± 2.8	5.5 ± 4.1	1.2 ± 2.5	2.5 ± 3.8
Coating on the pharyngeal wall	6.5 ± 2.3	7.0 ± 2.4	3.0 ± 4.0	2.3 ± 3.9
Pharyngeal transit time	4.8 ± 1.8	3.7 ± 2.3	1.8 ± 1.6	1.6 ± 2.2
Aspiration	4.2 ± 5.1	5.3 ± 4.3	3.3 ± 3.9	2.4 ± 3.0
Oral phase	20.7 ± 8.7	21.9 ± 7.8	15.5 ± 5.8	19.7 ± 5.5
Pharyngeal phase	35.0 ± 11.9	35.1 ± 9.9	18.0 ± 15.0	18.4 ± 11.8
Total	55.7 ± 13.7	57.0 ± 15.1	33.5 ± 18.9	38.1 ± 14.8

**TABLE 5:** The videofluoroscopic dysphagia scale (VDS) score before and after the intervention unilateral or bilateral neck dissection

	Unilateral neck dissection		Bilateral neck dissection	
	Before intervention	After intervention	Before intervention	After intervention
Lip closure	0.0 ± 0.0	0.2 ± 0.3	0.1 ± 0.2	0.4 ± 1.0
Bolus formation	3.3 ± 1.9	2.8 ± 1.0	3.3 ± 1.5	4.1 ± 1.3
Mastication	3.3 ± 1.7	5.3 ± 0.0	4.8 ± 1.7	5.1 ± 1.6
Apraxia	1.8 ± 1.0	1.5 ± 0.7	1.9 ± 0.9	2.3 ± 0.8
Tongue to palate contact	4.6 ± 2.5	3.3 ± 1.9	4.2 ± 2.7	6.1 ± 2.4
Premature bolus loss	1.8 ± 1.3	1.4 ± 1.1	1.6 ± 1.7	1.7 ± 1.6
Oral transit time	1.8 ± 1.0	1.5 ± 1.0	2.1 ± 0.8	2.6 ± 0.5
Triggering of pharyngeal swallowing	3.0 ± 1.2	2.6 ± 0.8	3.0 ± 1.3	2.9 ± 1.0
Vallecular residue	3.2 ± 1.8	2.2 ± 1.1	2.1 ± 1.2	1.9 ± 1.5
Laryngeal elevation	6.0 ± 4.2	5.3 ± 2.9	5.7 ± 3.1	6.8 ± 2.3
Pyriform sinus residue	3.8 ± 4.3	3.8 ± 4.7	2.1 ± 2.6	3.7 ± 4.0
Coating on the pharyngeal wall	3.8 ± 4.5	1.5 ± 1.7	4.6 ± 3.6	5.2 ± 4.3
Pharyngeal transit time	3.0 ± 2.6	2.0 ± 1.6	3.0 ± 2.2	2.5 ± 2.7
Aspiration	2.5 ± 3.0	3.0 ± 1.2	4.1 ± 4.7	3.8 ± 4.3
Oral phase	16.4 ± 7.7	16.0 ± 3.4	18.0 ± 7.5	22.3 ± 6.4
Pharyngeal phase	25.2 ± 18.9	20.3 ± 12.1	24.6 ± 15.8	26.8 ± 14.3
Total	41.6 ± 23.9	36.3 ± 13.9	42.7 ± 19.7	49.0 ± 17.7

## 5 DISCUSSION

Swallowing function is one of the major concerns in survivors of head and neck cancer.^12^ Dysphagia has been described as the most critical problem affecting the quality of life.^13^ We showed the effect of PAP on oral cancer using tongue pressure and VDS.

In this study, PAP improved tongue pressure and tongue to palate contact. Tongue pressure was a predictive factor for decreased oral and pharyngeal food residue, and it has been used for quantitative evaluation of oropharyngeal swallowing function.^4^ In addition, tongue pressure is associated with masticatory performance.^14^ The estimated weighted mean tongue pressure in healthy subjects using JMS is 39.3 ± 0.92 kPa and 30.3 ± 0.42 kPa in individuals under 60 years old and above 60 years old, respectively.^15^ In our study, 8 of 15 cases had undergone total or subtotal glossectomy. Tongue pressure without PAP was 6.6 ± 5.6 kPa, which was similar to the previous report; its value is 15.3% ± 5.6% decreased compared to before subtotal glossectomy.^16^ Palmer et al. reported that tongue pressure was positively correlated with suprahyoid muscle activities.^17^ Tongue pressure with PAP was significantly increased compared to without PAP. The wearing of PAP did not lead to a full recovery in tongue pressure compared with the normal population. However, the VDS score of the oral phase and the pyriform sinus residue were significantly improved with the PAP. It may be necessary to investigate not only the pressure, but also the timing, duration, and locations of the contacts between tongue and palate during the propulsion of a bolus from the oral cavity to the pharynx.^18^

The VDS scale using VF was developed to assess dysphagia's severity.^19^ This scale was originally created to quantify the severity of dysphagia of stroke patients,^20^ but there were also statistically significant correlations for other health conditions such as spinal cord injury, peripheral neuropathy, neurodegenerative disease, traumatic brain injury, brain tumor, poor general medical condition, and local structural lesions involving the head and neck.^21^ Moreover, the VDS allows clinicians to understand and explain dysphagia and delineate dysphagia's aggravation and improvement in detail as the scale consists of 14 items, including the oral and pharyngeal phases. In our study, total VDS score improved by 76.9% in the patients whose tongue pressure had improved. Weber et al. reported that 27.8% of patients did not improve in swallowing function with the PAP.^22^ These results suggest that PAP alone may not contribute to improved swallowing function in some patients. Hence, it is essential to combine PAP with other approaches, such as swallowing function training.^23^

Our study revealed that pyriform sinus residue was significantly improved after wearing PAP. Meyer et al. reported that only worsening oral and pharyngeal residue correlated considerably with the deteriorating quality of life of head and neck cancer survivors.^12^ They stated that oral and pharyngeal residue had an independent effect on patients' diet, willingness to eat in public, and ability to participate in social gatherings. The results of our study corroborate with findings from a previous study that showed that oral residue was reduced from 90% to 25% and pharyngeal residue from 25% to 10%.^24^ Increased pharyngeal pressure may have affected the reduction of the pyriform sinus residue. In the rat model, peaks of thyrohyoid electromyography bursts and oropharynx pressure were decreased following bilateral hypoglossal nerve transection, but significantly increased and were longer after covering the hard and soft palates with acrylic material.^25^

Furthermore, PAP may accelerate the laryngeal elevation in the upward direction with the upper esophageal sphincter (UES) opening. Intrabolus pressures are associated with relaxation of the upper esophageal sphincter.^26^ Posterior pharyngeal wall advancement increased to compensate for swallowing function among individuals with reduced tongue muscle strength.^27^ It is possible that the tongue pressure by PAP and the compensatory action of the pharynx increased the pharyngeal pressure and assisted in the dilatation of UES. Further studies are needed in order to evaluate pharyngeal pressure using manometry or other pressure measurements.

### 5.1 Limitation

There are a few limitations in this study that deserve mention. First, the timing of VF before and after PAP placement is different for each patient. Other factors, such as swallowing rehabilitation, may influence the outcome. Second, our study is a retrospective study with a small number of patients. Further prospective studies involving more patients are needed. Divide into a group that creates PAP before oral cancer surgery and a group that does not use it and compares the swallowing function after surgery.

## 6 CONCLUSION

The wearing of PAP can improve tongue pressure and tongue to palate contact, and show improvement in pyriform sinus residue. These results suggest a possible effect on the pharyngeal phase while wearing PAP.

## CONFLICT OF INTEREST

The authors declare no financial relationships or conflict of interest.

## AUTHORS' CONTRIBUTIONS

All authors had full access to the data in the study and take responsibility for the integrity of the data and the accuracy of the data analysis. *Conceptualization*, K.K., S.K., K.S.; *Data Curation*, I.K., T.H., T.K., J.O., R.Is.; *Project Administration*, I.K., R.Ik., J.S., A.H.‐K., J.O., A.O., N.Sh., K.K., S.K., K.S., Y.K.; *Methodology*, N.S., R.T.; *Investigation*, T.H., N.S., R.T., T.K., A.H.‐K.; *Formal Analysis*, T.H., R.Ik., J.S., R.Is.; *Resources*, A.O.; *Writing ‐ Original Draft*, R.Ik.; *Writing ‐ Review & Editing*, T.H., R.Ik., J.S., A.H.‐K., R.Is., N.Sh.; *Visualization*, I.K., R.Ik., R.T., J.O., R.Is.; *Supervision*, R.Ik., J.S., A.H.‐K., J.O., A.O., N.Sh., K.K., S.K., K.S., Y.K.; *Funding Acquisition*, Y.K.

## ETHICS STATEMENT

This study was approved by the Tohoku University Hospital Institutional Review Board (IRB protocol number: 2014‐1‐274). Consent was obtained from patients.

## Data Availability

The data that support the findings of this study are available from the corresponding author upon reasonable request.
